# First applications of a targeted exome sequencing approach in fetuses with ultrasound abnormalities reveals an important fraction of cases with associated gene defects

**DOI:** 10.7717/peerj.1955

**Published:** 2016-04-26

**Authors:** Constantinos Pangalos, Birgitta Hagnefelt, Konstantinos Lilakos, Christopher Konialis

**Affiliations:** 1 Genomis Ltd, London, United Kingdom; 2 InterGenetics–Diagnostic Genetic Centre, Athens, Greece; 3 Department of Haematology, “Laikon” General Hospital - University of Athens Medical School, Athens, Greece

**Keywords:** Exome sequencing, Fetal malformations, Prenatal diagnosis, Gene disorders

## Abstract

***Background.*** Fetal malformations and other structural abnormalities are relatively frequent findings in the course of routine prenatal ultrasonographic examination. Due to their considerable genetic and clinical heterogeneity, the underlying genetic cause is often elusive and the resulting inability to provide a precise diagnosis precludes proper reproductive and fetal risk assessment. We report the development and first applications of an expanded exome sequencing-based test, coupled to a bioinformatics-driven prioritization algorithm, targeting gene disorders presenting with abnormal prenatal ultrasound findings.

***Methods.*** We applied the testing strategy to14 euploid fetuses, from 11 on-going pregnancies and three products of abortion, all with various abnormalities or malformations detected through prenatal ultrasound examination. Whole exome sequencing (WES) was followed by variant prioritization, utilizing a custom analysis pipeline (*Fetalis* algorithm), targeting 758 genes associated with genetic disorders which may present with abnormal fetal ultrasound findings.

***Results.*** A definitive or highly-likely diagnosis was made in 6 of 14 cases (43%), of which 3 were abortuses (Ellis-van Creveld syndrome, Ehlers-Danlos syndrome and Nemaline myopathy 2) and 3 involved on-going pregnancies (Citrullinemia, Noonan syndrome, *PROKR2*-related Kallmann syndrome). In the remaining eight on-going pregnancy cases (57%), a *ZIC1* variant of unknown clinical significance was detected in one case, while in seven cases testing did not reveal any pathogenic variant(s). Pregnancies were followed-up to birth, resulting in one neonate harboring the *PROKR2* mutation, presenting with isolated minor structural cardiac abnormalities, and in seven apparently healthy neonates.

***Discussion.*** The expanded targeted exome sequencing-based approach described herein (*Fetalis)*, provides strong evidence suggesting a definite and beneficial increase in our diagnostic capabilities in prenatal diagnosis of otherwise chromosomally balanced fetuses with troubling ultrasound abnormalities. Furthermore, the proposed targeted exome sequencing strategy, designed primarily as a diagnostic rather than a research discovery tool, overcomes many of the problems and limitations associated with clinical wide-scale WES testing in a prenatal setting.

## Introduction

Recognizable fetal ultrasound (US) abnormalities are observed in approximately 3–5% of all pregnancies, while congenital abnormalities account for 20–25% of perinatal deaths (Centers for Disease Control and Prevention, 2008; Romosan et al., 2009). In everyday clinical practice, prenatal detection and prevention of severe congenital disorders is typically achieved through various levels of fetal ultrasonographic examination in the 1st or 2nd trimester of pregnancy; however, precise diagnosis of the underlying genetic defect is often challenging and elusive due to considerable clinical and genetic heterogeneity (Rice et al., 2011; Ermito et al., 2009; Schramm et al., 2009), while an exasperating factor is the lack in most cases of prior family history or other identifiable predisposing risks (Long & Sprigg, 1998).

In these cases, advances in molecular genetics nowadays offer a more detailed prenatal genetic investigation, mainly for chromosomal abnormalities revealed through array comparative genomic hybridization (aCGH), although the diagnostic yield from these studies typically does not exceed ∼10–15% (Konialis & Pangalos, 2015; Hillman et al., 2013), leaving a considerable residual risk for a potentially debilitating genetic disorder. Furthermore, genetic testing for gene mutations associated with the observed ultrasound abnormalities is limited to targeted testing of very few distinct genes suspected to be involved in the particular case and therefore comprehensive testing for gene disorders in the course of pregnancy has been highly selective and thus largely ineffective (Milunsky & Milunsky, 2015).

As an example, skeletal malformations and dysplasias, often detected through routine prenatal ultrasound examination, constitute a phenotypic finding in more than 300 syndromic and non-syndromic disorders and their genetic etiology may be due to a wide variety of genetic aberrations, ranging from copy number variations to single gene mutations. Similarly, fetal limb deformities, such as structural abnormalities of the fingers and toes (clinodactyly, polydactyly, etc.), are relatively frequent ultrasonographic findings and are also associated with a variety of syndromic and non-syndromic genetic disorders (Warman et al., 2011; Porter & Herman, 2011). The above may be expressed as dominant, recessive, or X-linked disorders or they may be the result of spontaneous *de novo* mutations, while many forms present with variable phenotypic expression and are more difficult to diagnose compared to lethal forms. Therefore, it is more than evident that fetal structural malformations and other fetal abnormalities revealed through prenatal ultrasound are characterized by considerable phenotypic and genetic heterogeneity. For example, single gene such as *DHCR7*, may be associated with a variety of ultrasound findings, which may include skeletal malformations and/or genitourinary abnormalities (e.g., hypospadias), typically associated with Smith-Lemli-Opitz syndrome. Similarly, other genes involved together with *DHCR7* in the cholesterol biosynthesis pathway, are also associated with a number of genetic disorders/syndromes (e.g., Antley-Bixler syndrome—*POR* gene), presenting with several common prenatal clinical manifestations (Porter & Herman, 2011).

As a rule, abnormal ultrasound findings require detailed genetic counseling, where the merits and limitations of available genetic testing options are discussed as well as the associated risks. However, an accurate diagnosis is a highly desirable prerequisite in this process, not only for parental decisions regarding the outcome of the current pregnancy but also for providing accurate counseling for the risk for future pregnancies and this is even more important in families with *recurrent* fetal ultrasonographic findings, pointing to an underlying inherited genetic disease.

Very recently, a rather limited number of retrospective studies, utilizing whole genome sequencing (Talkowski et al., 2012) or whole exome sequencing (WES) in small cohorts of neonates and aborted fetuses with various ultrasound and/or post-mortem structural abnormalities (Carss et al., 2014; Drury et al., 2015; Alamillo et al., 2015; Westerfield et al., 2015), provided initial proof-of-principle of large-scale prenatal next generation sequencing (NGS). The results from these studies have been the subject of a recent review and debate (Filges & Friedman, 2015; Chitty, Friedman & Langlois, 2016), where both the merits and limitations of WES applications in malformed fetuses are presented. In addition, it appears that the majority of couples have a positive attitude towards prenatal WES testing, particularly when confronted with troubling fetal ultrasound findings (Kalynchuk et al., 2015).

We present our initial findings and follow-up results, derived from a custom designed targeted exome sequencing strategy, as applied prospectively (on-going pregnancy) and retrospectively (abortuses) in a cohort of 14 euploid fetuses, all presenting with various ultrasonographic anomalies. This approach may afford a timely diagnosis in the course of pregnancy, while overcoming many of the pitfalls associated with large–scale prenatal NGS. We also discuss the potential benefits, the challenges and future developments of this testing strategy, through our central view of maintaining a careful and desirable balance between an increase in diagnostic potential and the undesirable ‘grey zones’ in prenatal diagnosis.

## Materials and Methods

### Cases and samples

Prenatal cases included in this study (period 03/2015–9/2015, see Table 1) were referred to our center for diagnostic genetic testing from maternity hospitals or fetal medicine centers throughout Greece and where the invasive procedure was performed. All cases involved euploid fetuses ascertained by prior prenatal aCGH, performed as previously described (Konialis & Pangalos, 2015). The study is in compliance with the Helsinki Declaration and in all cases mandatory pre-test genetic counseling was provided initially by the referring physician and subsequently in-house, where all couples were counseled specifically on all aspects of the genomic testing to be performed, particularly as applied in prenatal diagnosis, and provided their informed consent.

**Table 1:** Reported fetal ultrasound findings, *Fetalis* exome sequencing results, disease diagnosis and pregnancy outcome.

Case^a^	Gestation week	U/S findings	Prior history^b^	Gene variants detected	Diagnosis - syndrome	Confirmation and/or pregnancy outcome
1	Abortus, 27wk	Multiple limb deformities	No	*EVC2* c.2776G > A (p.E926K) & c.707T > C (p.V236A), double heterozygous	Ellis-van Creveld syndrome (AR)	Parents carriers
2	Abortus, 22wk	IUGR, joint contractures, mild hydrocephalus, decreased fetal movements	Yes	*NEB* c.11060C > T (p.A3687V) & c.11333T > C (p.I3778T), double heterozygous	Nemaline myopathy (AR)	Parents carriers, both mutations present in previously affected fetus
3	Abortus, 18wk	Hypoplastic right antebrachium, wrist and phalangeal defects of right hand	No	*COL3A1* c.811C > T (p.R271X), het	Ehlers-Danlos syndrome IV (AD)	Paternally inherited, mutation present in affected uncle
4	12wk	NT 4.8 mm and cystic hygroma in 1st trimester screen	No	*PTPN11* c.181G > A (p.D61N), heterozygous	Noonan syndrome (AD)	*de novo* mutation, known pathogenic, pregnancy terminated
5	23wk	Brain MRI abnormalities	Yes	*ASS1* c.725C > T (p.T242I) & c.971G > T (p.G324V), double heterozygous	Citrullinemia (AR)	Parents carriers, pregnancy terminated, similar findings in previous pregnancy
6	22wk	Interventricular septum heart defect	No	*PROKR2* c.518T > G (p. L173R), heterozygous^d^	PROKR2-related Kallmann syndrome (AD)	Live birth, 5 mo, surgical correction of heart defect, no other abnormality
7	27wk	Rotation of the cerebellar vermis, enlargement of the cisterna magna	Yes	*ZIC1* c.1208C > A (p.S403Y), heterozygous	Association with Dandy-Walker malformation, VOUS	Maternally inherited, previous pregnancy terminated with identical findings, pregnancy subsequently terminated
8	22wk	IUGR, short nasal bone, short long bones, possible hypospadias	No	No pathogenic mutation	–	Live birth, 9 mo, no abnormality reported
9	22wk	Unilateral clinodactyly	No	No pathogenic mutation	–	Live birth, 6 mo, no abnormality reported
10	18wk	Right hand polydactyly	No	No pathogenic mutation	–	Live birth, 5 mo, slight deformity-pseudodactyly surgically corrected, no other abnormality
11	21wk	Hydronephrosis, echogenic bowel, brachymelia	No	No pathogenic mutation	–	Live birth, 5 mo, no abnormality reported
12	24wk^c^	Hydronephrosis	No	No pathogenic mutation	–	Live birth, 4 mo, no abnormality reported
13	24wk^c^	Short humerus and femur, echogenic bowel	No	No pathogenic mutation	–	Live birth, 4 mo, no abnormality reported
14	23wk	NT 4.2 mm in 1st trimester screen, cystic hygroma in 2nd trimester	No	No pathogenic mutation	–	Live birth, 3 mo, no abnormality reported

**Notes.**

a Cohort 1-abortuses: cases 1–3. Cohort 2-on-going pregnancies: cases 4–14.

b Prior pregnancies with similar ultrasound findings.

c Twin pregnancy.

d Parents as yet unavailable for testing.

The samples consisted of either amniotic fluid (AF) or chorionic villi sampling (CVS) and were accompanied by our prenatal testing requisition form, stating the clinical indications (ultrasound findings and/or abortus autopsy reports). Parental peripheral blood samples were also collected for confirmatory analysis and/or follow-up testing.

### Whole exome libraries

Genomic DNA was isolated using the QIAamp DNA Blood Mini Kit (Qiagen, Valencia, CA, USA), quantified by the Qubit 2.0 fluorometer (Life Technologies, Thermo Fisher Scientific, Waltham, MA, USA), assessed on the Agilent TapeStation 2,200 (Agilent Technologies Inc., USA) and ∼120 ng was subsequently subjected to whole exome DNA library construction using the Ion AmpliSeq Whole Exome RDY (Life Technologies, Thermo Fisher Scientific, Waltham, MA, USA) essentially as described in the manufacturer’s protocol, with barcode incorporation.

AmpliSeq HiQ libraries for sequencing were prepared on the Ion OneTouch 2 system, quantified using the Qubit 2.0 fluorometer (Life Technologies, Thermo Fisher Scientific, Waltham, MA, USA) and massive parallel sequencing (MPS) was subsequently performed on the Ion Proton System utilizing a PI chip (Life Technologies, Thermo Fisher Scientific, Waltham, MA, USA), with 2 or 3 samples (in cases of family trio) loaded per run.

### Data analysis

Primary sequence data analysis was performed using Torrent Suite (4.2.0) with default parameters and variant calling was performed using the Ion Torrent Variant Caller (TSVC) plug-in (version 4.2-8-r87740) using default settings and relative to NCBI37/hg19 reference assembly. The resulting variants (vcf file) were annotated through the Ion Reporter 4.4 or 5.0 variant annotation analysis function and the annotated variants were subsequently imported for filtering, prioritization and evaluation into a local Exome Management Application (*EMA*) custom pipeline.

### Gene selection and variant prioritization—*Fetalis* algorithm

We selected an extensive panel of 758 genes (Table S1) associated with genetic disorders, which may present with fetal structural abnormalities detectable in the 1st, 2nd or 3rd trimester by ultrasound and/or other fetal monitoring techniques (e.g., echocardiogram, MRI, etc.). Mitochondrial DNA genes are not included. The list of genes and disorders was carefully compiled and manually curated using data from various sources (OMIM, 2016; Borrelli et al., 2008; Stenson et al., 2009; Bell et al., 2011; Saunders et al., 2012; GeneTests, 2015; Milunsky & Milunsky, 2015; Sabbagha, Tamura & Sabbagha, 2015; HPO database - Köhler et al., 2014), including the HPO-driven built-in function of the *EMA* pipeline. The *Fetalis* 758 genes and variant prioritization algorithm (C Konialis & Z Agioutantis, 2014, unpublished data) was specifically designed for hierarchical evaluation of gene variants, based on up to three user-selectable main ultrasonographic clinical findings, utilizing imported Human Phenotype Ontology (HPO) terminology (Köhler et al., 2014) and Phenomizer data (Köhler et al., 2009, http://compbio.charite.de/phenomizer/). In several instances, where HPO and/or Phenomizer terms and gene associations were not available, these were constructed and incorporated into the *EMA-Fetalis* pipeline database. The *Fetalis* pipeline is coupled to other built-in standard filtering functions in the *EMA* pipeline, such as mutation pathogenicity score, variant population frequency (1000 GP, NHLBI Exome Variant Server-EVS, ExAC consortium, local Greek variant database), etc. Final interpretation of results was performed manually, on the basis of the hierarchy ranking of the gene variants from the *EMA*-*Fetalis* algorithm, clinical experience and generally in accordance with published guidelines (Richards et al., 2015). Specifically in cases where clinical reports were made available in an *on-going pregnancy*, we decided to evaluate and report variants as having clinical significance *only* when falling into the following two categories: (a) known pathogenic mutations, previously reported in the literature and/or in mutation databases, and (b) ‘obligatory pathogenic’ variants, of the type nonsense, frameshift, indels, canonical splice-site variants (±2 intron donor–acceptor splice-sites). In selected cases (see Results), other type of variants, not included in any of the above categories (e.g., AR disorders—combination in a single gene of one known pathogenic mutation and one highly-likely pathogenic mutation), were also evaluated and reported (Richards et al., 2015). Standard Sanger DNA sequencing was used for variant confirmation and segregation analysis.

## Results

Prenatal cases were referred and considered for testing based on the type and severity of the reported fetal ultrasound findings or other fetal abnormalities observed post-mortem.

Specifically for on-going pregnancies, during pre-test counseling and prior to providing their informed consent, couples were informed that testing is targeted, that it will not reveal or report incidental findings and that it will only evaluate and report variants/mutations included in the aforementioned categories (see Materials and Methods).

In order to be able to provide a timely diagnosis in on-going pregnancies, we evaluated and subsequently applied the use of the remaining DNA sample, extracted directly from uncultured amniotic fluid or CVS samples, following previous prenatal aCGH testing. As the Ion Ampliseq Exome RDY reagent typically requires no more than 100–130 ng of input DNA, this type of library preparation coupled to MPS on the Ion Proton PI chip afforded a rapid protocol, not necessitating cell culture and able to deliver interpretable results in less than 10 days, including confirmatory analysis. All 14 fetal samples yielded whole exome libraries within acceptable quality criteria. The technical analysis parameters from each sample are provided in Table S2. On average, a total of approximately 2,200 ± 50 variants per sample/case were detected corresponding to the 758 genes (∼1,030 variants occurring in exons, 1–3 variants in splice sites); following gene prioritization (based on the reported ultrasonographic abnormalities for each case) and variant filtering through the *Fetalis* algorithm, approximately 1–3 potentially significant variants/mutations were left for manual inspection and final clinical evaluation, with no incidental findings. As an example, applying the *Fetalis* algorithm in Case 7 (see Table 1), a total of 243 exonic and splice-site variants were identified in 134 genes (out of the 758 *Fetalis* genes), prioritized due to their association with genetic disorders which may possibly include abnormalities of the cerebellar vermis and/or with Dandy-Walker malformation. Further filtering and prioritization through the *EMA-Fetalis* built-in parameters for: (a) variant frequency against our local Greek variant database, (b) for variant frequency against international databases (1000 GP, EVS, ExAc) and (c) for pathogenicity score and variant class, resulted in 2 potentially significant missense variants/mutations requiring final manual curation and clinical evaluation (c.1375C > T–p.R459C in the *CHD7* gene and the c.1208C > A–p.S403Y in the *ZIC1* gene). The *CHD7* gene missense mutation was quickly dismissed based on several factors (e.g., disease-phenotype association and relatively common allele frequency in ExAc), while the maternally-inherited *ZIC1* missense variant was at the time reported as a VOUS (see note added in proof).

Even allowing for Sanger sequencing confirmation and segregation analysis, final results were available in less than 10 days, with the potential of attaining an even faster turn-around time (<5 days).

The *Fetalis* targeted (758 genes) exome sequencing strategy provided overall a clinical diagnosis in 5 of 14 cases (36%, Table 1, cases 1–5), involving 3 products of abortion (Cohort 1, cases 1–3) and 2 on-going pregnancies (Cohort 2, cases 4 and 5). In one further on-going pregnancy case (Table 1, case 6), a highly-likely diagnosis of *PROKR2*-related autosomal dominant Kallmann syndrome was made, involving a known pathogenic mutation. Also, in another on-going pregnancy (Table 1, case 7), a novel probably pathogenic missense variant/mutation was detected in the *ZIC1* gene, which was maternally inherited and was reported as a variant of unknown clinical significance (VOUS). In the remaining 7 on-going-pregnancies (Table 1, cases 8–13), *Fetalis* testing did not reveal a known or ‘obligatory’ pathogenic mutation (see Materials and Methods) in the fetus, possibly associated with the reported ultrasound abnormalities.

### Cohort 1 - abortuses

In Case 1, a 24 year old G2P0 + 1 female was referred for genetic counseling following termination of pregnancy at 27 weeks of gestation. A prior US examination revealed a male fetus with bilateral limb abnormalities (polydactyl, short hand, brachydactyly) and talipes equinovarus. A histopathological examination of the abortus had not been performed. The results revealed the presence of 2 heterozygous missense variants: c.2776G > A (p.Glu926Lys) and c.707T > C (p.Val236Ala), in exon 7 and 17, respectively, of the *EVC2* gene. The variants are not present in dbSNP, 1000 GP, EVS Variant Server, while they are both present in the ExAc database with allele frequencies of 1.647e−05 and 2.48e−05, respectively. The variant c.2776G > A was predicted as possibly damaging, while the c.707T > C variant, occurring at the first base of exon 7, had a predicted non-pathogenic effect in terms of the amino-acid substitution (valine to alanine); however, a variety of *in silico* tools predicted to affect proper splicing of exon 7 through disruption of the acceptor splice-site. Subsequent Sanger sequencing of the parents determined that each was heterozygous for one of the variants, the c.707T > C variant originating from the mother and the c.2776G > A originating from the father, thus confirming compound heterozygosity and autosomal recessive inheritance. Mutations in the *EVC2* gene are known to be the cause of autosomal recessive Ellis-van Creveld syndrome (EVC, OMIM 225500) and autosomal dominant Weyers acrodental dysostosis (MIM 193530), rare malformation syndromes with a number of common phenotypic features, which include limb malformations (e.g., syndactyly, polydactyly) (Mankin, Jupiter & Trahan, 2011; Shen et al., 2011). Thus, the two mutations of the EVC2 gene detected in this case, combined with the reported US findings in the fetus provide an almost unequivocal diagnosis of EVC, and novel mutations with variable expressivity are very often uncovered in newly investigated patients (D’Asdia et al., 2013).

Cases 2 and 3 (Table 1) also involved abortuses with several US abnormalities, which had prompted the couples to terminate the pregnancy. In Case 2, with IUGR, joint contractures, mild hydrocephalus and decreased fetal movements, the fetus was found to be compound heterozygous for two missense variants/mutations of the *NEB* gene: a novel c.11060C > T (p.Ala3687Val) variant/mutation not present in public databases and a c.11333T > C (p.Ile3778Thr) variant/mutation present in the ExAc database with an allele frequency of 9.113e−05, both predicted as probably pathogenic. The parents were heterozygous-carriers for the mutations (c.11060C > T maternal, c.11333T > C paternal), while testing of a remaining fetal DNA sample from the previous similarly affected pregnancy confirmed the presence of both mutations in that fetus as well. Mutations in the *NEB* gene are associated with autosomal recessive Nemaline myopathy 2 (OMIM 2560), a congenital form of which may present with US findings during pregnancy, similar to those reported in this fetus (OMIM, 2016; Lammens et al., 1997; Ryan et al., 2001). In Case 3, a hypoplastic right antebrachium, wrist and phalangeal defects of the right hand of the fetus were reported, with no other major post-mortem autopsy findings. Testing revealed the heterozygous presence of a nonsense *COL3A1* c.811C > T (p.R271X) mutation, associated with Ehlers-Danlos syndrome IV (AD), an autosomal dominant disorder with variable expressivity. Among the various clinical symptoms associated with the disease, limb malformations (e.g., hypoplastic limb and limb reduction) have been reported in a subset of patients (Pepin, Murray & Byers, 2015; Pepin et al., 2014). Genetic testing in the extended family revealed that the mutation was present in the father and his brother (paternal uncle), the latter presenting also with various limb deformities and limb reductions. It is worth noting that null *COL3A1* mutations (nonsense mutations) are reported to present with reduced penetrance and with atypical clinical symptoms, most of these confined to vascular and limb anomalies (Pepin et al., 2014; Leistritz et al., 2011).

### Cohort 2 - on-going pregnancies

In this cohort of on-going pregnancy cases, *Fetalis* testing was performed as part of prenatal risk assessment, requested by the couples and the attending obstetrician, following genetic counseling.

Case 4, involving a primagravida, the reported US abnormalities in the 12th week of pregnancy were elevated nuchal translucency (*NT* = 4.8 mm) and cystic hygroma. Initial prenatal aCGH testing was negative, while subsequent *Fetalis* testing revealed the heterozygous presence of a known pathogenic *PTPN11* mutation c.181G > A (p.Asp61Asn) (rs397507510, Tartaglia et al., 2002) not present in either parent (*de novo*). Mutations in the *PTPN11* gene are associated with the autosomal dominant genetic disorder Noonan syndrome 1 (OMIM 163950), typically presenting with elevated NT and/or cystic hygroma in the 1st trimester ultrasound. The couple decided to terminate the pregnancy and the findings from a subsequent post-mortem histological analysis confirmed the presence of the disease in the male fetus.

Case 5, a G2P0 woman, was referred with fetal MRI abnormalities (Dandy-Walker malformation, ventricular dilatation) also observed in the previous pregnancy (terminated). *Fetalis* testing revealed that the female fetus was compound heterozygous for the *ASS1* gene variants/mutations: c.725C > T (p.Thr242Ile)—maternal and c.971G > T (p.Gly324Val)—paternal, the latter being a known pathogenic ASS1 gene mutation (Engel, Höhne & Häberle, 2009). The maternal c.725C > T (p.Thr242Ile) variant/mutation is not previously reported in any public databases or in the literature and was characterized as probably pathogenic (75% *EMA* score).

Mutations in the *ASS1* gene are associated with autosomal recessive citrullinemia (OMIM 215700), which often presents with congenital brain abnormalities detectable by MRI (Majoie et al., 2004). Following genetic counseling, the couple decided to terminate the pregnancy.

Case 6 involved a fetus with ventricular septal heart defect as the sole US finding and aCGH testing (mainly for exclusion of DiGeorge syndrome) was negative. The couple and their obstetrician requested further testing through *Fetalis*, where it was found that the fetus harbored a heterozygous *PROKR2* c.518T > G (p. L173R) known pathogenic mutation, previously reported in several Kallmann syndrome patients (Sarfati et al., 2010; Sarfati et al., 2013), frequently presenting with septal heart defects. The mother received detailed counseling regarding this finding (father unavailable) and decided to continue with the pregnancy. The requested follow-up information regarding the newborn reported routine surgical correction of the heart defect and at the age of five months there are as yet no other phenotypic abnormalities.

Case 7 represented a fetus from a G2P0 woman, with reported US and brain MRI abnormalities of rotation of the cerebellar vermis and enlargement of the cisterna magna. The fetus from the previous pregnancy was also reported with identical findings. The *Fetalis* test revealed the presence of a heterozygous missense mutation c.1208C > A (p.S403Y) of the *ZIC1* gene, a gene possibly associated with Dandy-Walker malformation (OMIM 220200). Subsequent confirmatory Sanger sequencing revealed that the mutation was also present in the mother and the variant was reported as a VOUS; however, the family decided to terminate the pregnancy. A post-mortem examination of the fetus was not performed.

Finally, in the seven remaining on-going pregnancy cases (Table 1, Case 8–Case14), *Fetalis* testing, requested as part of risk assessment for various fetal US abnormalities, did not reveal the presence of variants/mutations which, according to the test criteria (see Materials and Methods), could be characterized as disease-causing. For all these pregnancies, following post-test genetic counseling, the couples decided to continue the pregnancy, resulting in the birth of seven apparently healthy newborns, presently at the age of 3–5 months old.

## Discussion

The identification of the underlying genetic cause in fetuses with US abnormalities, especially in non-consanguineous families, is a challenging task and prenatal genetic diagnosis is typically limited to the investigation of possible chromosomal imbalances, either through conventional fetal karyotype analysis or, more recently, through aCGH. However, as aCGH will only uncover the underlying genetic cause in <15% of these cases (Konialis & Pangalos, 2015; Hillman et al., 2013), an accurate diagnosis is rarely achieved and the necessary advice regarding the precise risks associated with the current pregnancy as well as recurrence risk in subsequent pregnancies is elusive and relies on empirical knowledge. Therefore, the decision to terminate the pregnancy is based solely on the type of fetal ultrasound findings and how the associated risks for a possible severe disorder are perceived by the couple.

In this report we describe the first application of *Fetalis*, a targeted 758 genes exome sequencing approach, in three products of abortion (Cohort 1) and prospectively in 11 on-going pregnancies (Cohort 2), all with diverse abnormal ultrasound findings and of a troubling but non-extreme type, often encountered in routine prenatal diagnosis. Notwithstanding known and unavoidable technical limitations inherent to NGS testing (e.g., incomplete coverage), a highly-likely or definitive diagnosis was achieved in all three abortuses and in three out of 11 on-going pregnancies, resulting in an overall diagnosis rate of ∼36–43%. Although this figure is obviously a very preliminary assessment from a limited number of cases, it nonetheless provides an initial proof-of-principle regarding the merits of the approach described herein. Most importantly, follow-up data of apparently healthy newborns, born out of on-going pregnancy cases where *Fetalis* was performed as part of prenatal risk assessment, confirmed the high degree of reassurance provided from the negative results of the test.

There are several important issues we wish to address and discuss, regarding the possible clinical use of the *Fetalis* testing strategy, as described in this report. Although there is no doubt that WES in fetuses with ultrasound abnormalities is in principle expected to provide a considerable increase in our current prenatal diagnostic capabilities, albeit with many caveats (Chitty, Friedman & Langlois, 2016), the limited data from the few recent studies (Carss et al., 2014; Drury et al., 2015; Alamillo et al., 2015; Westerfield et al., 2015) and from our own initial efforts provide valuable insights to several important limitations regarding its clinical utility in a prenatal setting. Firstly, interpretation of the results following wide scale WES or WGS testing is hampered by the sheer complexity of data analysis and the concomitant difficulties and time required for variant prioritization and final clinical evaluation. Secondly, analysis of WES data will inevitably lead to the uncovering of a large number of incidental findings, unrelated to the reported US findings, leading to serious counseling and ethical issues. The third and most important point is related to the clinical evaluation of numerous WES variants of unknown significance, a very common occurrence in these types of studies.

The targeted exome testing strategy described in this report overcomes many of these limitations and concerns. The volume of data (variants) derived from the 758 genes is an order of magnitude less than the corresponding WES data and this fact, coupled to the rapid laboratory protocol and the custom-developed *EMA-Fetalis* prioritization pipeline, affords a highly cost-effective, more simplified and timely diagnosis (even <1 week) during the course of pregnancy. In addition, the decision to clinically evaluate and report in an on-going pregnancy only known pathogenic variants or ‘obligatory’ pathogenic variants (see Materials and Methods) reduces dramatically the number of incidental findings (see Results) and the reporting of dubious variants of unknown significance (VOUS), thus simplifying both pre- and post-test genetic counseling. All the above become even more important, if not paramount, when investigating fetuses with less severe US abnormalities, a common troubling occurrence and request in prenatal diagnosis. Although one may argue that the primary purpose and value of prenatal exome sequencing lies in the investigation of highly pathological US anomalies (Filges & Friedman, 2015), in everyday clinical practice this is not common as both the obstetrician and the parents wish to ascertain whether a less severe US finding (e.g., ambiguous genitalia) may only be ‘the tip of the iceberg,’ hiding underneath other much more serious phenotypic features, not yet apparent through US examination of the developing fetus. In such a case, through precise knowledge of the diseases excluded following our targeted testing approach, a negative test result provides a highly desirable reassurance.

To further illustrate the aforementioned points, one could perhaps compare our approach to the application of a targeted aCGH, as opposed to a high-resolution aCGH, in prenatal diagnosis (Konialis & Pangalos, 2015; Ahn et al., 2014; Oneda et al., 2014; Ganesamoorthy et al., 2013). The *Fetalis* targeted exome sequencing strategy could be viewed as a targeted aCGH, focusing on regions of known pathogenicity, similar to the implementation of ‘focused’ lower resolution prenatal aCGH (Ahn et al., 2014). Although it may miss certain abnormalities, possibly picked-up by a more in-depth WES analysis, it has the advantage of avoiding: (a) VOUS, (b) extensive family studies or costly WES-trio analysis, (c) complicated genetic counseling and perhaps these drawbacks cannot be offset by the possibly higher detection rate.

Finally, the *Fetalis* testing strategy is flexible and its diagnostic yield will definitely increase, without compromising clarity. Although the *Fetalis* pipeline is currently targeting and evaluating variants detected only in the 758 genes, the initial step in the analysis involves construction of a whole exome (WES) library and therefore whole-exome variant data are readily available. Hence, as we gain more detailed knowledge, the pipeline may continuously incorporate new genes and pathogenic variants, identified through postnatal WES investigations in severely affected newborns and children.

## Conclusions

There is little doubt that prenatal exome sequencing has the potential of becoming soon a routine diagnostic tool. However, as it is applied in a very sensitive and vulnerable setting, we should take the necessary care and precautions so that we do not extend its limits to the point where it might become more of a problem and less relevant and focused to the questions it was set out to resolve.

To our knowledge, this is the first report exploiting the diagnostic potential of a novel targeted and well-defined exome sequencing strategy, which may be deployed rapidly in a clinical prenatal setting and particularly in the course of pregnancy, with minimal ambiguous results and incidental findings. Although the number of cases presented in this report is too small to afford an accurate figure relating to the diagnostic yield, to this end we have initiated an extensive collaborative study with the aim of resolving questions relating to the diagnostic potential of our approach across different categories of US abnormalities, such as heart malformations, skeletal dysplasias, etc. We believe that the overall *Fetalis* strategy and the initial data presented in this report provide a sound, affordable and encouraging basis for routine clinical implementation.

NOTE ADDED IN PROOF: Since the submission of the manuscript, new developments have emerged regarding Case 7 (see Results and Table 1). The couple and their attending obstetrician informed us recently that a new (third) pregnancy is currently at the 23rd week of gestation, with identical fetal ultrasound brain MRI anomalies as the previous two pregnancies and requested clinical genetic evaluation. Suspecting a possible involvement of the maternal *ZIC1* gene variant, we suggested an immediate brain MRI examination of the mother, where it was revealed that she presents with the same structural brain malformations detected in all three fetuses. We subsequently performed a first clinical genetic evaluation of the mother, where a mild Crouzon syndrome-like phenotype was noted, with mild dysmorphic features and irregular head shape. At the time of the initial *Fetalis* results assessment of Case 7 in May 2015, there had been no published reports directly linking *ZIC1* gene variants to a recognizable pathological phenotype and thus, in accordance with our testing and reporting guidelines, the maternally inherited *ZIC1* gene variant was reported as a VOUS. However, a recent publication (Twigg et al., 2015) has now established an association between *ZIC1* gene mutations and coronal craniosynostosis accompanied with structural brain malformations, very similar to those observed in all three fetuses and the mother of Case 7. Further to this recent report, a more detailed clinical evaluation of the mother and of the current pregnancy is under way. We believe that this recent development provides further supporting evidence regarding the specificity and clinical validity of our approach described herein.

## Supplemental Information

10.7717/peerj.1955/supp-1 Table S1 Fetalis 758 genes list

10.7717/peerj.1955/supp-2 Table S2 NGS coverage analysis metrics Whole exome sequencing coverage analysis metrics for each case.

## Additional Information and Declarations

### Data Availability

The following information was supplied regarding data availability: The raw sequencing data is too large to be made available online. Please consult the Fetalis variants data for each case (Case 1 to Case 14):
https://figshare.com/s/548f76f45727396447cf. The gene list, current at the time of this article’s submission, is available as Table S1. This list is constantly reviewed and updated so the updated version can be requested from Christopher Konialis, genetics@otenet.gr.
