# Supplementary material for: First applications of a targeted exome sequencing approach in fetuses with ultrasound abnormalities reveals an important fraction of cases with associated gene defects
Source: PeerJ. 2016 Apr 26;4:e1955. doi: 10.7717/peerj.1955 (PMC4860337; doi:10.7717/peerj.1955)
Supplement: Table S1 [file peerj-04-1955-s001.pdf]

Supplementary Table S1: Fetalis 758 genes

#	GENE	#	GENE	#	GENE
1	AAAS	46	ASPA	91	CASK
2	ABAT	47	ASPM	92	CBL
3	ABCC6	48	ASS1	93	CC2D2A
4	ABCD1	49	ASXL1	94	CCBE1
5	ABCD3	50	ATL1	95	CCDC28B
6	ACAN	51	ATM	96	CCM2
7	ACO2	52	ATP6V0A2	97	CD96
8	ACTA1	53	ATP7A	98	CDC6
9	ACTB	54	ATP8A2	99	CDH1
10	ACTG1	55	ATR	100	CDKL5
11	ADAMTS10	56	ATRX	101	CDKN1C
12	ADAMTS17	57	ATXN10	102	CDON
13	ADAMTSL2	58	B3GALNT2	103	CDT1
14	ADAR	59	B3GALT6	104	CEP164
15	ADGRG1	60	B3GALTL	105	CEP290
16	ADSL	61	B3GAT3	106	CEP41
17	AHI1	62	B3GNT1	107	CEP57
18	AHI1	63	B4GALT1	108	CFTR
19	AIMP1	64	B9D1	109	CHAT
20	AIPL1	65	B9D2	110	CHD7
21	AIRE	66	BBIP1	111	CHMP1A
22	AKR1C2	67	BBS1	112	CHN1
23	AKT1	68	BBS10	113	CHRNA1
24	AKT3	69	BBS12	114	CHRNB1
25	ALDH1A3	70	BBS2	115	CHRND
26	ALMS1	71	BBS4	116	CHRNE
27	ALPL	72	BBS5	117	CHRNG
28	ALX1	73	BBS7	118	CHST14
29	ALX3	74	BBS9	119	CHST3
30	ALX4	75	BCOR	120	CHSY1
31	AMER1	76	BDNF	121	CHUK
32	AMT	77	BIN1	122	CKAP2L
33	ANKRD11	78	BMP2	123	CNTN1
34	ANTXR2	79	BMP4	124	COG4
35	AP4B1	80	BMPER	125	COL11A1
36	AR	81	BMPR1B	126	COL11A2
37	ARFGEF2	82	BRAF	127	COL18A1
38	ARHGAP31	83	BRIP1	128	COL1A1
39	ARID1A	84	BSND	129	COL1A2
40	ARID1B	85	BUB1	130	COL2A1
41	ARL13B	86	BUB1B	131	COL3A1
42	ARL6	87	BUB3	132	COL4A1
43	ARVCF	88	C12ORF57	133	COL5A1
44	ARX	89	C5ORF42	134	COL5A2
45	ASNS	90	CACNA1A	135	COL6A1

Supplementary Table S1: Fetalis 758 genes

#	GENE	#	GENE	#	GENE
136	COL6A2	181	DYM	226	FANCM
137	COL6A3	182	DYNC1H1	227	FAT4
138	COL7A1	183	DYNC2H1	228	FBN1
139	COL9A1	184	EARS2	229	FBN2
140	COL9A2	185	EBP	230	FBXL4
141	COLEC11	186	ECEL1	231	FGD1
142	COMT	187	EFNB1	232	FGF10
143	COX7B	188	EFTUD2	233	FGF17
144	CPT2	189	EGR2	234	FGF8
145	CRB1	190	EHMT1	235	FGF9
146	CREBBP	191	EIF2AK3	236	FGFR1
147	CRH	192	EIF4A3	237	FGFR2
148	CRLF1	193	EMD	238	FGFR3
149	CRX	194	EMG1	239	FH
150	CSPP1	195	EMX2	240	FIG4
151	CTCF	196	EOGT	241	FKBP14
152	CTNS	197	EP300	242	FKRP
153	CUL7	198	EPG5	243	FKTN
154	CXCR4	199	EPHX1	244	FLNA
155	CYP11B1	200	ERBB3	245	FLNB
156	CYP17A1	201	ERCC1	246	FLRT3
157	CYP19A1	202	ERCC2	247	FLT4
158	CYP21A2	203	ERCC4	248	FLVCR2
159	CYP2U1	204	ERCC5	249	FOXC1
160	DARS	205	ERCC6	250	FOXC2
161	DCHS1	206	ERLIN2	251	FOXE1
162	DCX	207	ESCO2	252	FOXG1
163	DDHD2	208	EVC	253	FRAS1
164	DDX59	209	EVC2	254	FREM1
165	DHCR24	210	EYA1	255	FREM2
166	DHCR7	211	EZH2	256	FTO
167	DHH	212	FA2H	257	FUZ
168	DHODH	213	FAM111A	258	G6PC3
169	DIS3L2	214	FAM123B	259	GAA
170	DKC1	215	FAM20C	260	GATA1
171	DLL3	216	FAM58A	261	GATA4
172	DLX5	217	FANCA	262	GATA6
173	DMD	218	FANCB	263	GBA
174	DMPK	219	FANCC	264	GBA2
175	DNM2	220	FANCD2	265	GBE1
176	DOCK6	221	FANCE	266	GCSH
177	DOK7	222	FANCF	267	GDF1
178	DPYD	223	FANCG	268	GDF3
179	DSP	224	FANCI	269	GDF5
180	DUSP6	225	FANCL	270	GDF6

Supplementary Table S1: Fetalis 758 genes

#	GENE	#	GENE	#	GENE
271	GFAP	316	HYLS1	361	KIF14
272	GFM1	317	IBA57	362	KIF1A
273	GJA1	318	ICK	363	KIF2A
274	GJB2	319	IDS	364	KIF5C
275	GJC2	320	IDUA	365	KIF7
276	GLDC	321	IER3IP1	366	KISS1R
277	GLE1	322	IFIH1	367	KMT2D
278	GLI2	323	IFT172	368	KRAS
279	GLI3	324	IFT27	369	L1CAM
280	GLUL	325	IFT80	370	L2HGDH
281	GMPPB	326	IFT88	371	LAMA2
282	GNAI3	327	IGBP1	372	LAMB1
283	GNAO1	328	IGHMBP2	373	LAMC3
284	GNPTAB	329	IHH	374	LARGE
285	GNPTG	330	IKBKG	375	LBN
286	GP1BB	331	IL17RD	376	LBR
287	GPC3	332	IMPDH1	377	LCA5
288	GPC6	333	INPP5E	378	LEMD3
289	GPI	334	INSR	379	LFNG
290	GPR56	335	IQCB1	380	LHB
291	GPSM2	336	IRF6	381	LHX3
292	GRHL3	337	ISPD	382	LHX4
293	GRIP1	338	ITGA6	383	LIFR
294	GRM1	339	ITGA8	384	LMBR1
295	GUCY2D	340	ITGB4	385	LMNA
296	H19	341	ITPR1	386	LMNB1
297	HADHA	342	JAG1	387	LMX1B
298	HADHB	343	JAM3	388	LRAT
299	HCCS	344	JUP	389	LRP2
300	HDAC8	345	KAL1	390	LRP4
301	HES7	346	KANSL1	391	LZTFL1
302	HESX1	347	KAT6B	392	MAP2K1
303	HIBCH	348	KCNA1	393	MAP2K2
304	HIRA	349	KCNJ13	394	MAP3K1
305	HOXA11	350	KCNJ2	395	MASP1
306	HOXA13	351	KCNK9	396	MBTPS2
307	HOXA2	352	KCNQ1OT1	397	MCOLN1
308	HOXD13	353	KCNQ2	398	MCPH1
309	HPGD	354	KCNT1	399	MED12
310	HRAS	355	KCTD1	400	MEF2C
311	HS6ST1	356	KCTD7	401	MEGF10
312	HSD17B3	357	KDM6A	402	MEOX1
313	HSD17B4	358	KIAA0196	403	MESP2
314	HSPG2	359	KIAA1279	404	MFRP
315	HYAL1	360	KIAA2022	405	MGP

Supplementary Table S1: Fetalis 758 genes

#	GENE	#	GENE	#	GENE
406	MID1	451	NSDHL	496	PIGV
407	MIPOL1	452	NSMF	497	PIK3CA
408	MKKS	453	NT5C2	498	PIK3R2
409	MKS1	454	OBSL1	499	PIP5K1C
410	MLH1	455	OCLN	500	PITX1
411	MOCS1	456	OCRL	501	PKHD1
412	MOCS2	457	OFD1	502	PLCB4
413	MPL	458	OPHN1	503	PLEC
414	MPZ	459	ORC1	504	PLK4
415	MRPS16	460	ORC4	505	PLOD1
416	MRPS22	461	ORC6	506	PLOD3
417	MSH2	462	OTX2	507	PLP1
418	MSH6	463	PAFAH1B1	508	PMM2
419	MSX1	464	PALB2	509	PMP22
420	MSX2	465	PAX2	510	PMS2
421	MTM1	466	PAX3	511	PNKP
422	MUSK	467	PAX6	512	POLR1C
423	MVK	468	PCNT	513	POLR1D
424	MYBPC1	469	PDE4D	514	POLR3A
425	MYH2	470	PDE6D	515	POMGNT1
426	MYH3	471	PDGFB	516	POMGNT2
427	MYH8	472	PDGFRB	517	POMK
428	NAA10	473	PDHA1	518	POMT1
429	NBN	474	PDHX	519	POMT2
430	NDE1	475	PDYN	520	POR
431	NEB	476	PEX1	521	PORCN
432	NEK1	477	PEX10	522	POU1F1
433	NF1	478	PEX11B	523	PQBP1
434	NFIX	479	PEX12	524	PRDM5
435	NIN	480	PEX13	525	PRG4
436	NIPBL	481	PEX14	526	PRKAR1A
437	NKX2-5	482	PEX16	527	PROK2
438	NMNAT1	483	PEX19	528	PROKR2
439	NODAL	484	PEX2	529	PROP1
440	NOG	485	PEX26	530	PRRX1
441	NOTCH2	486	PEX3	531	PRSS56
442	NPC1	487	PEX5	532	PRX
443	NPC2	488	PEX6	533	PSAP
444	NPHP1	489	PEX7	534	PSAT1
445	NPHP3	490	PFKM	535	PTCH1
446	NPR2	491	PGM1	536	PTCH2
447	NR0B1	492	PHF6	537	PTDSS1
448	NR5A1	493	PHF8	538	PTEN
449	NRAS	494	PIGA	539	PTH1R
450	NSD1	495	PIGL	540	PTPN11

Supplementary Table S1: Fetalis 758 genes

#	GENE	#	GENE	#	GENE
541	PVRL1	586	RTTN	631	SMC1A
542	PYCR1	587	RUNX2	632	SMC3
543	RAB18	588	RYR1	633	SMOC1
544	RAB23	589	SACS	634	SMS
545	RAB3GAP1	590	SALL1	635	SNAP29
546	RAB3GAP2	591	SALL4	636	SNIP1
547	RAB40AL	592	SAMHD1	637	SOS1
548	RAD21	593	SATB2	638	SOX10
549	RAD51C	594	SC5D	639	SOX2
550	RAF1	595	SCARF2	640	SOX3
551	RAI1	596	SDCCAG8	641	SOX9
552	RAPSN	597	SEC23A	642	SPATA7
553	RAX	598	SEMA3A	643	SPECC1L
554	RB1	599	SEMA3E	644	SPG11
555	RBM10	600	SEPN1	645	SPRED1
556	RBM8A	601	SEPSECS	646	SPRY4
557	RD3	602	SEPT9	647	SPTAN1
558	RDH12	603	SETBP1	648	SRD5A2
559	RECQL4	604	SF3B4	649	SRD5A3
560	RELN	605	SH3PXD2B	650	SRY
561	RET	606	SHANK3	651	STAC3
562	RIPK4	607	SHH	652	STAMBP
563	RIT1	608	SHOC2	653	STAT3
564	RMND1	609	SIX1	654	STS
565	RMRP	610	SIX3	655	STXBP1
566	RNASEH2A	611	SIX5	656	SUFU
567	RNASEH2B	612	SIX6	657	SUMO1
568	RNASEH2C	613	SKI	658	SYNE1
569	RNU4ATAC	614	SLC12A1	659	TACR3
570	ROR2	615	SLC12A6	660	TAF2
571	RPE65	616	SLC20A2	661	TAZ
572	RPGRIP1	617	SLC25A19	662	TBC1D20
573	RPGRIP1L	618	SLC26A2	663	TBX1
574	RPL11	619	SLC2A10	664	TBX15
575	RPL15	620	SLC35A2	665	TBX22
576	RPL26	621	SLC35A3	666	TBX3
577	RPL35A	622	SLC35D1	667	TBX4
578	RPL5	623	SLC6A8	668	TBX5
579	RPS10	624	SLC9A6	669	TBX6
580	RPS17	625	SLX4	670	TCF4
581	RPS19	626	SMAD3	671	TCOF1
582	RPS24	627	SMAD4	672	TCTN1
583	RPS26	628	SMARCA4	673	TCTN2
584	RPS6KA3	629	SMARCB1	674	TCTN3
585	RPS7	630	SMARCE1	675	TECPR2

Supplementary Table S1: Fetalis 758 genes

#	GENE	#	GENE
676	TECT1	721	UBE3B
677	TFAP2A	722	UFD1L
678	TGFB3	723	UPF3B
679	TGFBR1	724	UPK3A
680	TGFBR2	725	UTRN
681	TGIF	726	VANGL1
682	TGIF1	727	VANGL2
683	TMCO1	728	VAX1
684	TMEM138	729	VIPAS39
685	TMEM216	730	VPS13B
686	TMEM231	731	VPS33B
687	TMEM237	732	VSX2
688	TMEM5	733	VTI1A
689	TMEM67	734	WDPCP
690	TMEM70	735	WDR11
691	TNNI2	736	WDR19
692	TNNT3	737	WDR34
693	TNXB	738	WDR35
694	TP63	739	WDR60
695	TPM2	740	WDR62
696	TRAPPC9	741	WDR81
697	TREM2	742	WNT10B
698	TREX1	743	WNT3
699	TRIM32	744	WNT4
700	TRIP11	745	WNT5A
701	TRPV4	746	WNT7A
702	TSC1	747	WT1
703	TSC2	748	WWOX
704	TSEN34	749	ZBTB18
705	TSEN54	750	ZDHHC9
706	TSPYL1	751	ZEB2
707	TTC8	752	ZFPM2
708	TUBA1A	753	ZFYVE26
709	TUBA8	754	ZIC1
710	TUBB2B	755	ZIC2
711	TUBB3	756	ZMPSTE24
712	TUBGCP6	757	ZNF423
713	TULP1	758	ZNF469
714	TWIST		
715	TWIST1		
716	TYR		
717	TYROBP		
718	UBA1		
719	UBB		
720	UBE3A		

Supplementary Table S1: Fetalis 758 genes
